# Very-late-onset neuromyelitis optica spectrum disorder: a case report and review

**DOI:** 10.3389/fneur.2025.1559872

**Published:** 2025-04-10

**Authors:** Rui-Ting Liu, Yu-Kun Guo, Xia Du, Heng Li

**Affiliations:** ^1^Department of Neurology, Central Hospital Affiliated to Shandong First Medical University, Jinan, China; ^2^Cheeloo College of Medicine, Shandong University, Jinan, China

**Keywords:** NMOSD, very-late-onset, COVID-19 vaccine, immunotherapy, satralizumab

## Abstract

Neuromyelitis optica spectrum disorder (NMOSD) is a rare autoimmune disorder that causes demyelination within the central nervous system, typically manifesting as symptoms of optic neuritis and myelitis. We report the case of a 92-year-old patient with NMOSD who was admitted to our hospital; hers is currently the oldest reported case of NMOSD globally. The onset occurred after COVID-19 vaccination, and the patient responded well to treatment with satralizumab.

## Introduction

Neuromyelitis optica spectrum disorder (NMOSD) is a rare inflammatory demyelinating disorder of the central nervous system. It usually presents as a clinical syndrome or magnetic resonance imaging (MRI) findings related to the optic nerve(s), spinal cord, area postrema or other brainstem areas, the diencephalon or the cerebrum (1). Serum aquaporin-4 immunoglobulin G (AQP4-IgG) is considered to be its pathogenic antibody and, thus, is widely used in clinical diagnostic practice (2). A previous analysis showed that the overall mean age of onset was 38.3 years, with ages ranging from 2 to 86 years in AQP4-associated NMOSD (3). Here, we report a case of a 92-year-old woman who presented with longitudinally extensive transverse myelitis after a COVID-19 vaccination and was diagnosed with NMOSD.

## Case description

**Figure 1:**
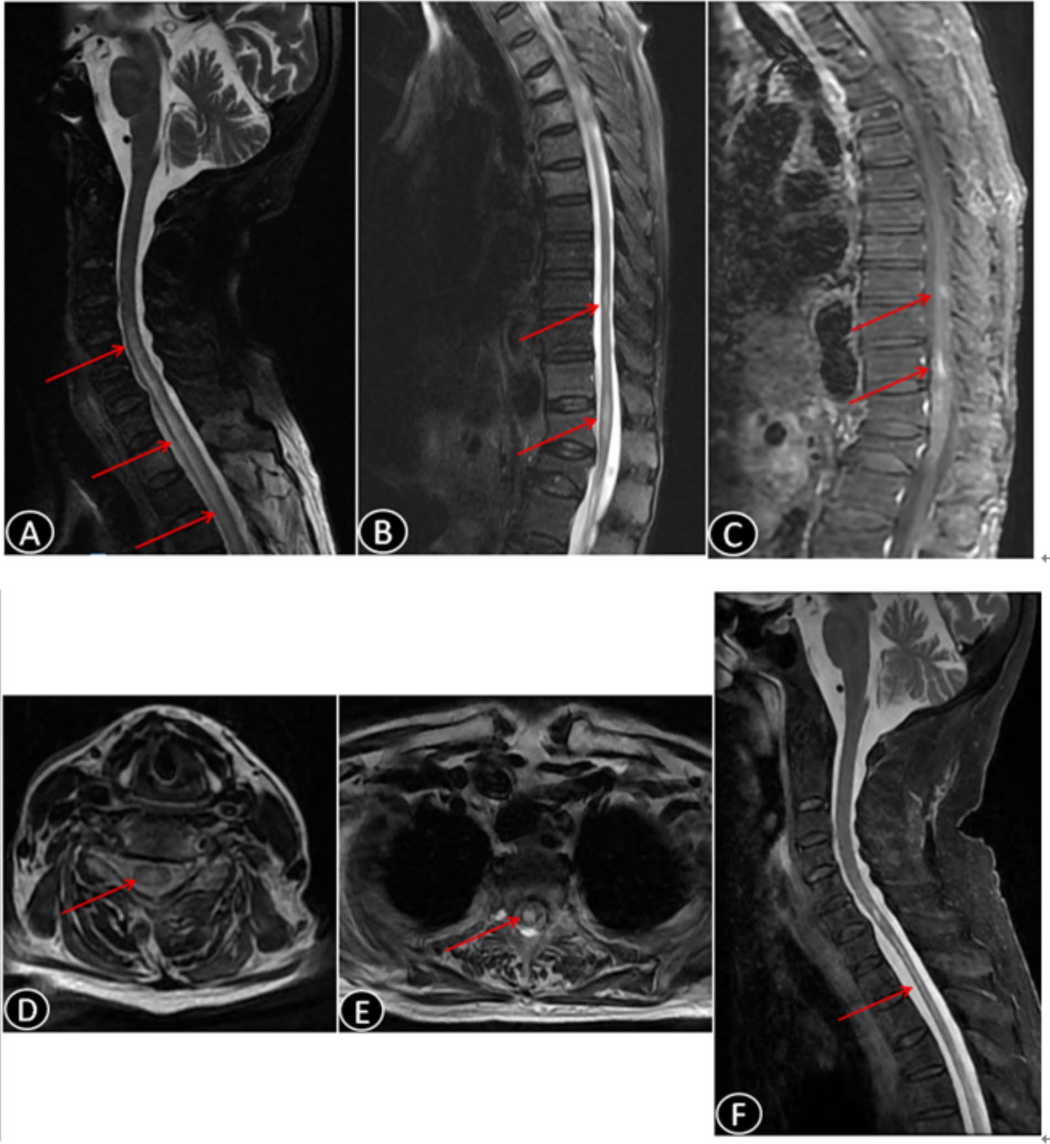
Magnetic resonance images (MRIs) showing the patient’s neuromyelitis optica spectrum disorder lesions. **(A,B)** Sagittal MRI of the spinal cord showing patchy T2 fat-compression hyperintensity at vertebral levels C4–T4, T9 and T11–12, along with slight swelling of the spinal cord at C4–T4. **(C)** Sagittal MRI of the spinal cord showing patchy enhancement at vertebral levels T9, 11–12. **(D,E)** Transverse section of T2-weighted MRI of the spinal cord, revealing an abnormal hyperintense signal located in the center of the spinal cord. **(F)** Sagittal T2-weighted MRI of the spinal cord showing a significant decrease in the abnormal high signals from C5 to T12, with resolution of the spinal cord edema and notable atrophy of the spinal cord during the follow-up of approximately 11 months after the diagnosis.

A 92-year-old Chinese female was admitted to the Department of Neurology of our hospital on October 24, 2023, due to a fever having lasted for 3 days and weakness in both lower limbs for 8 h, accompanied by urinary retention, without significant neck or chest pain. One week prior to admission, she had a COVID-19 vaccination. She had had hypertension, an old cerebral infarction (without sequelae) and mild coronary heart disease for years, but she was able to care for herself in daily life. Upon admission, her physical examination revealed: a temperature of 36.7°C, pulse of 69 beats per minute, respiratory rate of 20 breaths per minute and blood pressure of 150/78 mmHg. Auscultation of the heart and lungs was unremarkable. Neurological examination revealed that she was fully conscious and able to speak clearly, with grade 5 muscle strength in both upper limbs, grade 5− in the left lower limb and grade 4 in the right lower limb. There was decreased pinprick sensation below the T5 level, and reduced vibration sensation in the right lower limb. Bilateral positive findings were observed for Chaddock’s sign, and positive Babinski reflex was found in the right lower limb. Signs of meningeal irritation were negative. The patient was tested on the Mini-Mental State Examination and Montreal Cognitive Assessment, scoring 23 (maximum score: 30) and 16 (maximum score: 30), respectively. MRI of the spinal cord showed abnormal signals in the long segments of the spinal cord at C4 to T4, T9 and T11–T12, with partial enhancement, as shown in Figures 1A–E. The cranial diffusion-weighted image revealed a new infarction lesion in the left thalamus, with no other significant abnormalities observed (image not shown). Cerebrospinal fluid analysis revealed a white blood cell count of 28 × 10^6^/L (0–8 × 10^6^/L) and protein level of 0.64 g/L (0.15–0.45 g/L). Tests for demyelination-related antibodies showed a positive serum anti-AQP4 antibody (1:320, collagen binding assay method), a negative serum anti-myelin oligodendrocyte glycoprotein and negative oligoclonal bands in both serum and cerebrospinal fluid. Based on the patient’s medical history, physical examination and additional tests, a diagnosis of NMOSD was made.

Two days after admission, she gradually developed an intestinal obstruction and experienced a decrease in lower limb muscle strength to grade 1–2. Treatment began on day 3 with intravenous (IV) immunoglobulin (0.4 g/kg/day) for 5 days, followed by IV methylprednisolone from day 7. Given the advanced age of the patient, we started with methylprednisolone 500 mg/day for 3 days, then halved the dosage every 3 days until reaching 60 mg/day, which was administered orally. Calcium, potassium and stomach-protection medications were also administered. Cyclophosphamide was used to suppress the immune response and prevent relapse. Over the next 2 months, she developed *Klebsiella pneumoniae* pneumonia and a *Candida albicans* urinary infection, leading to the discontinuation of the cyclophosphamide. After anti-infection treatment, the patient recovered.

**Figure 2:**
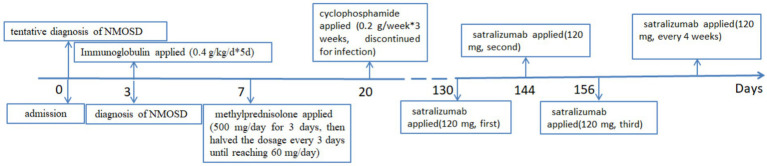
The patient’s entire treatment sequence, since her admission with a diagnosis of neuromyelitis optica spectrum disorder (NMOSD).

Given her age and susceptibility to infections, the patient was started on satralizumab in March 2024, to prevent a relapse. At that time, she had an Expanded Disability Status Scale (EDSS) score of 8, on a scale where the most-severe disability is rated as a “10.” Three subcutaneous injections of 120 mg of satralizumab were administered at weeks 0, 2, and 4. Afterwards, a maintenance dose of 120 mg was administered every 4 weeks. The timeline of the patient’s treatments is shown in Figure 2. Subsequent evaluations of liver function, platelet counts and neutrophil levels revealed no significant abnormalities. After 4 months, the patient demonstrated the ability to stand with support from a wall and her EDSS score improved to 7. In a follow-up in September 2024, the repeat MRI of the cervical spinal cord showed a decrease in abnormal signals, resolution of spinal cord edema and the appearance of spinal cord atrophy, as shown in Figure 1F. The patient discontinued the use of steroids in September 2024, and has been continuously administered satralizumab alone. Under continuous observation, there has been no recurrence so far.

## Discussion

The prevalence range of NMOSD is approximately 0.5 ~ 10/100,000 in various regions of the world and is more prevalent in non-Caucasian populations (4). In 2020, China released data on the incidence rate of NMOSD, with an overall incidence of 0.278/100,000, and incidences of 0.075/100,000 and 0.347/100,000 in children and adults, respectively, based on a nationwide mandatory database of the Hospital Quality Monitoring System (5). A meta-analysis showed that the mean age of onset of AQP4-IgG-positive NMOSD was 41.7 years, and that the age of onset was expected to increase with the increase in the life span of the overall population (3). In a literature search on PubMed, the highest age of onset found for NMOSD is 88 years old (6). In the present case, the 92-year-old patient was admitted to the hospital with symptoms of spinal cord lesions. Spinal cord MRI revealed long-segment myelitis, and serum AQP4-IgG was positive, leading to a diagnosis of NMOSD. This is currently the highest known age of onset for an NMOSD patient. Some researchers classify NMOSD with an onset age of <50 years as early-onset NMOSD (EO-NMOSD); that with an onset age of ≥50 years as late-onset NMOSD (LO-NMOSD); and that with an onset age of ≥70 years as very-late-onset NMOSD (VLO-NMOSD) (7, 8). Compared with patients with EO-NMOSD, those with LO- or VLO-NMOSD exhibit: more-severe disability; faster progression; severe myelitis with longitudinal spinal cord involvement, rather than optic neuritis (ON); higher AQP4-IgG seropositivity and higher AQP4-IgG titers; more white matter hyperintensities; more-severe sleep disorders; higher levels of anxiety; poorer cognitive function; and higher scores on the Clinical Dementia Rating-community affairs scale (9–12). It has also been reported that peripapillary hemorrhages with optic disc edema may be a feature that distinguishes VLO-NMOSD-ON from demyelinating ON (13). The elderly patient described in this article exhibited long-term spinal cord pathology without any visual impairment, which is consistent with previous research (9–12).

The most common adverse events with the COVID-19 vaccine are injection site pain, fever, fatigue, etc. (14). In recent years, there has been a proliferation of research on the occurrence of neuroimmunological diseases such as NMOSD following COVID-19 vaccination, yet the relationship between them and the mechanisms remain unclear (15–17). Because most studies originate from single centers with a limited number of reported cases and are retrospective observational research with some incomplete data (17, 18). Although the pathogenesis is unknown, research has revealed that human antigens and severe acute respiratory syndrome coronavirus 2 (SARS-CoV-2) molecules may exhibit similar molecular properties, which may cause autoimmune diseases in vaccine recipients. The cross-reactivity of 21 human tissue antigens with antibodies to SARS-CoV-2 provides a potential explanation for the observed autoimmunity caused by SARS-CoV-2 mRNA vaccines, which affects various systems, including the gastrointestinal, cardiovascular and nervous systems (19).

Worse prognosis and severer motor disability in LO-NMOSD may be attributed to more extensive inflammation and impaired reparation mechanism (20). So the managing immunosuppression in elderly patients faces severe challenges. Several new biological agents with development strategies based on the mechanism of NMOSD have been evaluated in clinical trials. For example, inebilizumab targets antibody-producing B cells (21), eculizumab targets complement protein C5 (22), and satralizumab block interleukin-6 (IL-6) signaling (23). Aging is associated with elevated levels of circulating cytokines and pro-inflammatory markers (24), alongside a decline in immune system function (8). Studies have shown that serum levels of interleukin-6 (IL-6) increase with age (25), and IL-6 is implicated in the initiation and progression of cytokine storms (26). IL-6 serves as a crucial signal for the IL-6 amplifier, which is one of the main mechanisms driving inflammation, particularly chronic inflammation (27). Satralizumab is a novel monoclonal antibody drug that primarily acts as an IL-6 receptor antagonist, modulating and reducing inflammatory responses by inhibiting the IL-6 signaling pathway, thereby exerting therapeutic effects. Research has demonstrated that patients with VLO-NMOSD may experience therapeutic benefit from administration of high-dose IV methylprednisolone (28). However, compared with patients with EO-NMOSD, patients with VLO-NMOSD face a significantly increased risk of adverse reactions to high-dose steroid pulse therapy, primarily due to preexisting conditions such as hypertension and diabetes. Therefore, vigilant monitoring throughout the treatment process is crucial. In the present case, the patient experienced multiple infections during the initial stages of the disease, necessitating particular caution when implementing sequential treatment. Given the patient’s advanced age, sequential therapy with satralizumab was administered although both clinical trials of satralizumab included patients aged ≤74 years (23, 29). Over a subsequent follow-up period, the patient exhibited no signs of relapse, demonstrated an improved EDSS score, and laboratory tests revealed no significant abnormalities. These findings suggest that satralizumab is a safe treatment option for individuals with VLO-NMOSD. The use of satralizumab in our patient has also expanded its applicable population. The follow-up of this patient is ongoing. Aging is associated with elevated levels of circulating cytokines and pro-inflammatory markers (24), alongside a decline in immune system function (8). Studies have shown that serum levels of interleukin-6 (IL-6) increase with age (25), and IL-6 is implicated in the initiation and progression of cytokine storms (26). IL-6 serves as a crucial signal for the IL-6 amplifier, which is one of the main mechanisms driving inflammation, particularly chronic inflammation (27). Satralizumab is a novel monoclonal antibody drug that primarily acts as an IL-6 receptor antagonist, modulating and reducing inflammatory responses by inhibiting the IL-6 signaling pathway, thereby exerting therapeutic effects. Research has demonstrated that patients with VLO-NMOSD may experience therapeutic benefit from administration of high-dose IV methylprednisolone (28). However, compared with patients with EO-NMOSD, patients with VLO-NMOSD face a significantly increased risk of adverse reactions to high-dose steroid pulse therapy, primarily due to preexisting conditions such as hypertension and diabetes. Therefore, vigilant monitoring throughout the treatment process is crucial. In the present case, the patient experienced multiple infections during the initial stages of the disease, necessitating particular caution when implementing sequential treatment. Given the patient’s advanced age, sequential therapy with satralizumab was administered although both clinical trials of satralizumab included patients aged ≤74 years (23, 29). Over a subsequent follow-up period, the patient exhibited no signs of relapse, demonstrated an improved EDSS score, and laboratory tests revealed no significant abnormalities. These findings suggest that satralizumab is a safe treatment option for individuals with VLO-NMOSD. The use of satralizumab in our patient has also expanded its applicable population.

This patient has been taking satralizumab for 11 months now, with no recurrence and no serious adverse events, including major infections. We should continue with follow-up observations to assess the long-term effectiveness of satralizumab. Although our experience with satralizumab is encouraging, larger studies on satralizumab in elderly NMOSD patients are clearly needed to confirm its benefits.

## Conclusion

The immune mechanism underlying the onset of VLO-NMOSD may differ from that in EO-NMOSD. Given that elderly patients often have multiple comorbidities, caution is needed in the follow-up treatment after acute-phase therapy. Satralizumab, an IL-6 inhibitor, may be a good option for elderly patients with VLO-NMOSD.

## Data Availability

The original contributions presented in the study are included in the article/supplementary material, further inquiries can be directed to the corresponding author.
